# Upper Gastrointestinal Bleeding Secondary to an Incidental, Impacted Foreign Body in the Duodenum

**DOI:** 10.7759/cureus.6971

**Published:** 2020-02-12

**Authors:** Emuobor Odeghe, Azeberoje Osueni, Opeyemi O Owoseni, Funmi Adeniyi, Olufunmilayo Lesi

**Affiliations:** 1 Gastroenterology, University of Lagos and Lagos University Teaching Hospital, Lagos, NGA; 2 Medicine, Brookdale University Hospital and Medical Center, Brooklyn, USA; 3 Gastroenterology, Federal Medical Center, Abeokuta, NGA; 4 Pediatrics/Gastroenterology, College of Medicine, University of Lagos, Lagos, NGA; 5 Gastroenterology, Lagos University Teaching Hospital, Lagos, NGA

**Keywords:** endoscopy, foreign body, upper gastrointestinal bleeding, duodenum, stomach, melena, ulceration

## Abstract

A 68-year-old woman presented with a three-week history of upper abdominal discomfort, vomiting of coffee ground substance, and passage of tarry stools. There were no typical risk factors for gastroduodenal or liver disease. Gastroscopy done showed a fishbone impacted in the wall of the pyloric opening with its free end abutting on the wall of the duodenum resulting in a duodenal ulcer. Antral erosions were also noted. Retrieval forceps were used to retrieve the fishbone. The patient did not remember eating any fish containing meal, and there was no odynophagia. This case emphasizes the importance of considering foreign bodies as a cause of upper gastrointestinal bleeding as well as the need for an endoscopic review of all patients with upper gastrointestinal bleed.

## Introduction

Ingestion of foreign bodies that lodge in the upper gastrointestinal tract is common. Most objects can pass through the gastrointestinal tract spontaneously [1]. Approximately 80% of ingested foreign bodies that reach the stomach will pass through the gastrointestinal tract uneventfully, while the remaining 20% may cause complications such as obstruction, perforation, or hemorrhage [2]. We report the case of a 68-year-old woman who presented with upper abdominal discomfort, two episodes of vomiting of coffee grounds, and passage of dark, tarry stools, which were later found to be due to upper gastrointestinal bleeding from an incidental, impacted foreign body in the duodenum.

## Case presentation

**Figure 1:**
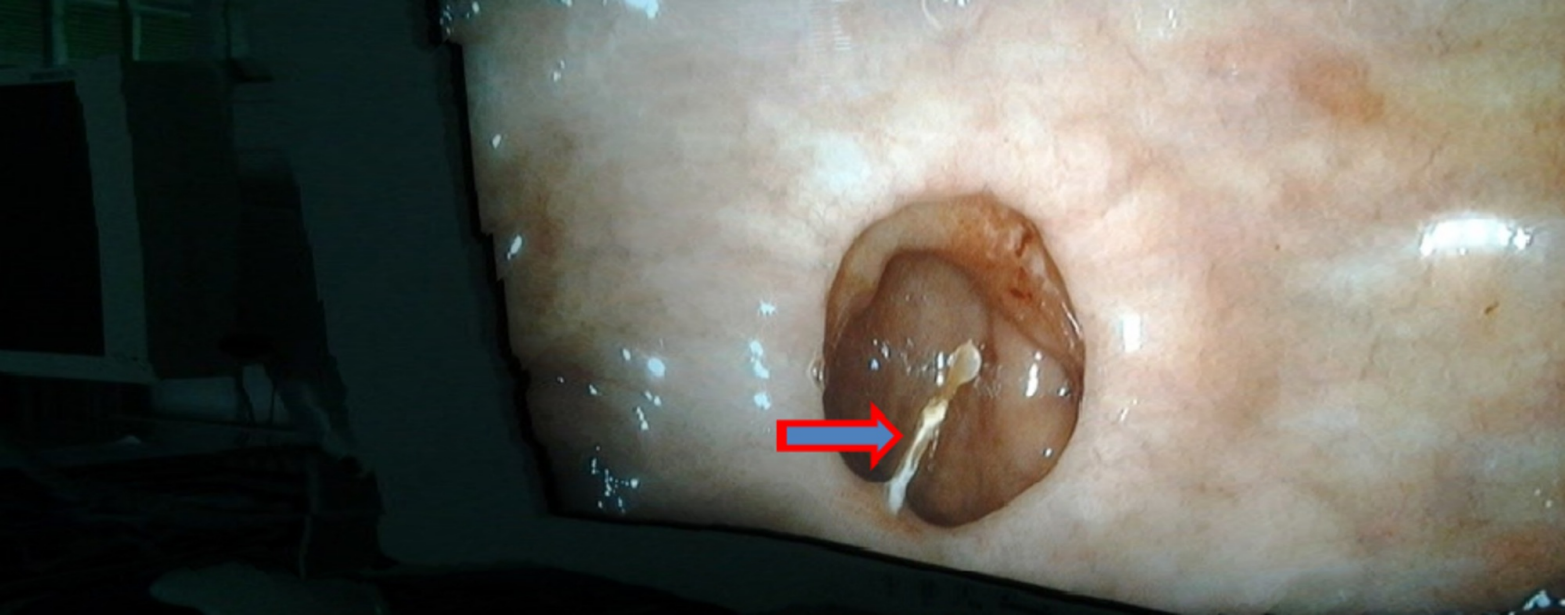
Endoscopic image showing the fishbone in the first part of the duodenum, with one end embedded in the wall and the other end abutting the opposite wall of the duodenum.

**Figure 2:**
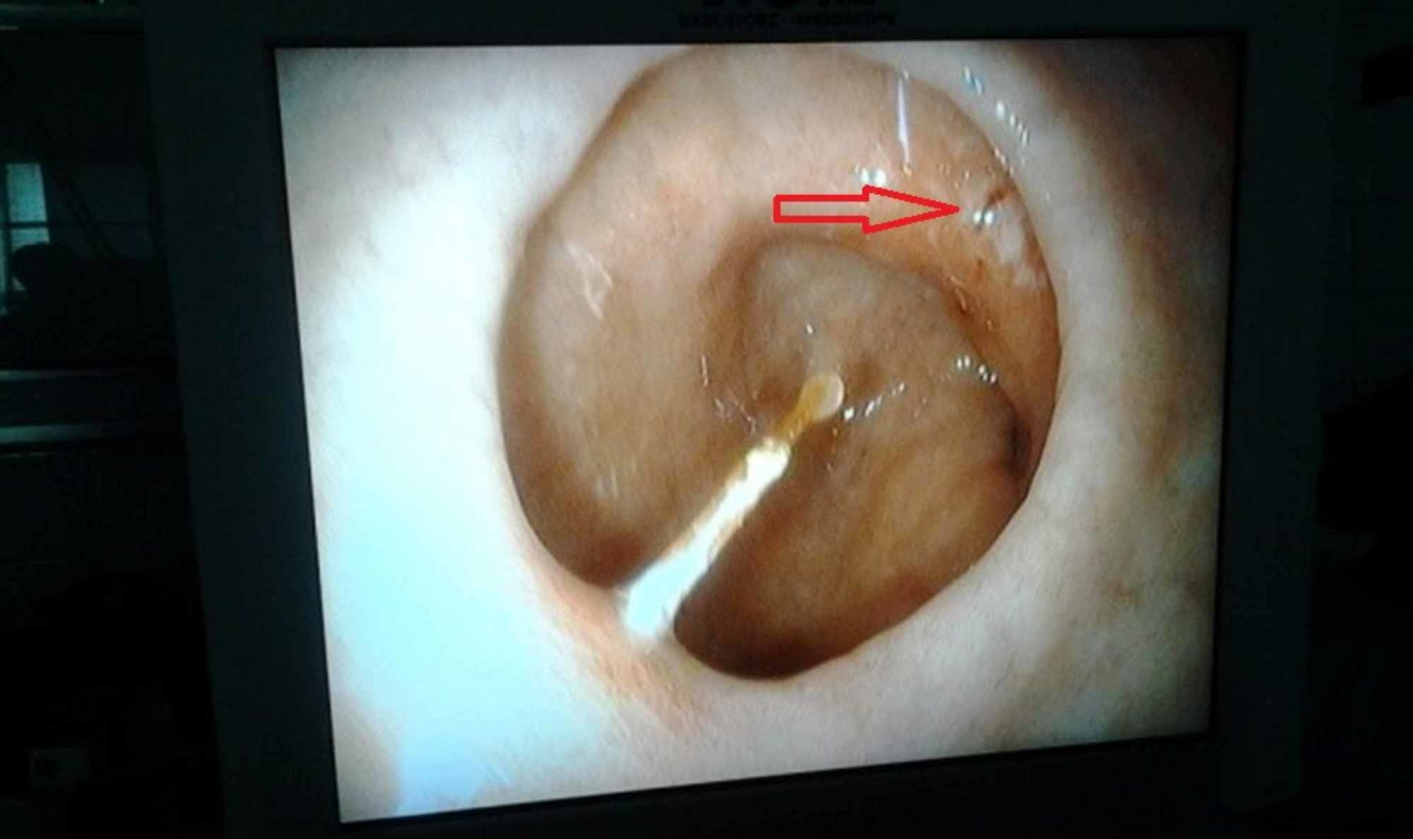
Image showing an ulcer in the first part of the duodenum caused by the fishbone.

A 68-year-old woman presented with a three-week history of upper abdominal discomfort, two episodes of coffee-ground emesis, and passage of dark, tarry stools. She did not take alcohol, non-steroidal anti-inflammatory drugs, or herbal concoctions. She had no history of heartburn, regurgitation, or any problems with swallowing. She had no previous diagnosis of liver disease and no symptoms suggestive of liver disease. Examination revealed an elderly woman, who was in no respiratory distress. She was generally well looking and not pale, also did not have jaundice. Vitals signs were normal. On abdominal examination, there was mild tenderness in the epigastrium; otherwise, it was unremarkable. Rectal examination revealed the presence of black tarry stool. Examination of the cardiovascular, respiratory, and nervous systems was unremarkable. She was mildly anemic with a hemoglobin of 9.2 g/dL, and other laboratory tests were within normal limits. The patient had an upper gastrointestinal endoscopy showing areas of petechial hemorrhage in the stomach. A foreign body (later identified as a 2.5 cm long fishbone) was found in the first part of the duodenum with one end embedded in the wall, and the other end abutting the opposite wall of the duodenum. The free end had produced a 2 cm long ulcer on the opposite wall. This ulcer most likely bled due to peristaltic contractions as the sharp end of the foreign body caused more damage (Figures 1, 2).

Retrieval forceps were used to successfully remove the embedded foreign body. On further questioning, the patient did not remember when she last ate a fish containing meal.

## Discussion

Ingestion of foreign bodies is common in clinical practice. Most objects pass through the gastrointestinal tract spontaneously, but some need endoscopic or surgical removal [3-5]. Factors that predispose to foreign body ingestion include male gender, alcohol or illicit drug use, use of dental prostheses, neuromuscular diseases, psychiatric illnesses, feeding disorders, and individuals in custody of prisons or orphanages [3,6]. The following gastrointestinal diseases may predispose to the presence of foreign bodies in the gastrointestinal tract: esophageal carcinoma, strictures, nutcracker esophagus, diverticulum, postgastrectomy, hiatus hernia, and achalasia [7,8]. The commonly encountered foreign bodies in adults include food boluses and pieces of food, fishbones, chicken bones, coins, and dental prostheses [7,9]. Sometimes, the offending object appears extremely unlikely such as guitarist pick and cat whisker [10,11].

It is vital to obtain an accurate history of the timing, type of ingested object, and onset of symptoms. Symptoms may include retrosternal discomfort, odynophagia, dysphagia, melena, hematemesis, and fatigue [12-17]. Patients may not remember when the foreign body was ingested, as in this patient, and this may delay diagnosis [18,19]. Time from the ingestion of the foreign body to the onset of symptoms may vary from days to weeks [4,5,12,18,19]. The risk of retention and subsequent perforation or ulceration by these ingested objects depends on the nature of the foreign body (sharp or blunt) as well as the anatomical variations in the gastrointestinal tract with congenital, physiological narrowing or angulations, and previous surgery increasing these risks [20].

Complications that may occur from the presence of foreign bodies in the upper gastrointestinal tract include perforation, ulcerations, upper gastrointestinal bleeding, retroperitoneal hemorrhage, peritonitis, and intra-abdominal abscess [4,12,14-20]. Our patient developed both ulceration and upper gastrointestinal bleeding. Management includes endoscopic retrieval of the object or surgical removal [7,13]. If bleeding is encountered during endoscopic removal of sharp/pointed objects, timely and meticulous resuscitation should be started with appropriate treatment (injection of adrenaline) if required at the bleeding point. A psychological evaluation may be necessary when the ingestion of a foreign body was intentional or in patients with suspected psychiatric illnesses or dementia [20].

## Conclusions

We present a rare case of unremembered foreign body (fishbone) ingestion causing upper gastrointestinal bleeding in a 68-year-old female. Clinicians must consider foreign bodies as a cause of upper gastrointestinal bleeding even though it is not present in the history provided by the patient. Endoscopic evaluation and subsequent retrieval of the foreign body may be required to prevent complications.
